# Supplementary material for: Non-invasive monitoring of adrenocortical function in female domestic pigs using saliva and faeces as sample matrices
Source: PLoS One. 2020 Jun 25;15(6):e0234971. doi: 10.1371/journal.pone.0234971 (PMC7316308; doi:10.1371/journal.pone.0234971)
Supplement: Attachment — Submitted filename: Response to reviewer.docx [file pone.0234971.s001.docx]

**Response to editor and reviewers**

Dear Dr Wolf,

Thank you for submitting your manuscript to PLOS ONE. After careful consideration, we feel that it has merit but does not fully meet PLOS ONE’s publication criteria as it currently stands. Therefore, we invite you to submit a revised version of the manuscript that addresses the points raised during the review process.

As suggested by Reviewer 1, the manuscript needs to be revised thoroughly before it can be considered for acceptance.

We look forward to receiving your revised manuscript.

Kind regards,

Govindhaswamy Umapathy, PhD
Academic Editor
PLOS ONE

Journal Requirements:

1. When submitting your revision, we need you to address these additional requirements.

Please ensure that your manuscript meets PLOS ONE's style requirements, including those for file naming. The PLOS ONE style templates can be found at

<http://www.journals.plos.org/plosone/s/file?id=wjVg/PLOSOne_formatting_sample_main_body.pdf> and <http://www.journals.plos.org/plosone/s/file?id=ba62/PLOSOne_formatting_sample_title_authors_affiliations.pdf>

2. In your Methods section, please include a comment about the state of the animals following this research. Were they euthanized or housed for use in further research? If any animals were sacrificed by the authors, please include the method of euthanasia and describe any efforts that were undertaken to reduce animal suffering

**We thank the editor for the opportunity to resubmit our revised manuscript. As suggested we have addressed the comments from the reviewers. We also added information concerning the state of the animals after this study was finished.**

Reviewers' comments:

Reviewer #1: The paper of Wolf et al (PONE-D-19-24903) aims at validating a method for evaluating adrenocortical activity via measuring faecal glucocorticoid metabolites (fGCMs) in pigs. The authors performed an ACTH challenge test (physiological validation) and a biological validation (transportation) in 6 female, immature pigs. They tested 4 different EIAs on a subset of sample (3 pigs) and found a 5α-pregnane-3β,11β,21-triol-20-one EIA best suited. Clear increases after ACTH injection and transportation in all 6 animals successfully validated the chosen EIA. An additional storage experiment demonstrated that FGCMs were stable up to 48 h. I think the experimental setup is well designed and the study adds knowledge about fGCMs analysis in an important species, where such methods have not been successfully validated so far. However, I miss a clear statement about the limitations/weaknesses of the study. The authors only used female, immature pigs. Because reproductive status (age) and sex are serious confounders in fGCM analyses, their influence needs to be elucidated (and the method validated in mature animals of both sexes) before a broad application of this method in pigs can be recommended.

**We thank the reviewer for the valuable input and we hope that our revised version sufficiently addresses the respective points raised.**

Detailed comments (ordered by appearance in the ms).
Title: I suggest indicating that only females (in female domestic pigs, or sows) were used (important as sex differences might be present). Better use “sample matrices” instead of “hormone matrices” (only metabolites are present in faeces)

**The title has been adjusted as suggested**

Abstract: line 24: Replace “In order of not adding” with “To avoid”.

**The sentence has been corrected (line 24)**

Line28: “sample matrices” (also line 80, 150)

**The sentence has been adjusted as suggested (lines 28, 77, 151)**

I suggest to reword: “An assay measuring faecal glucocorticoid metabolites (fGCM) with a 3ß,11ß-diol group has proven suited to determine adrenocortical activity , showing…” (the antibody is not against those metabolites).

**The sentence has been adjusted as suggested (line 28ff)**

Line 31: Better: A cortisol EIA was used… (the immunogen is cortisol-3-CMO:BSA)

**The sentence has been corrected (line 30ff)**

Lines 33-38: That could be described shorter (especially as collection and transport from distant locations might be a problem in wildlife, but unlikely in domestic pigs, or?)

**As suggested, the sentence has been shortened to assist the reader (although not common, longer transportation of domestic livestock occurs in SA) (line 35ff)**

Line 52: stereotypic

**The sentence has been corrected (line 52)**

Line 67: Your focus is domestic livestock – so would not be another review better suited here?
Somewhere in the introduction, it might be worth mentioning that pigs are an especially stress susceptible species.

**Alternative references have been provided. We also added a statement about the comparatively high stress susceptibility of pigs (line 47f)**

Line 85: ..on the stability of….

**The sentence has been corrected (line 84f)**

Line 90: I wonder when females of this breed get sexually mature, but 14 weeks old animals are too young.

**In this breed puberty is reached between 115 and 160 days and they usually deliver their first set of piglets at about one year of age.**

Line 94: normally written in italics and with 2 words: ad libitum

**The sentence has been corrected (line 94)**

Line 103: 50 IU (10 µg) there is a space between a number and its dimension (many other cases too – e.g. line 134; 138, etc..).

**We apologize for this oversight and added respective spaces between given numbers and their dimensions throughout the manuscript.**

Line 104: Add country! Switzerland?

**We added the country of origin (Switzerland)( line 105)**

Line 106: Were samples from all defecations collected?

**Samples were taken as per scheduled timelines throughout the experiment as described in line 118ff. During the indicated period, samples from all voided faeces were collected (line 119)**.

Here and elsewhere: I suggest to replace the hyphen with a word space.

**Corrected**

Line 114: Salivette, or?

**Yes, corrected (line 116)**

Line 117-118: I guess the portion was taken from the core of each samples, and then mixed? Or why is it important to take it from the core of a well-mixed sample?

**We apologize for the confusion. Yes, the portion was taken from the core of a sample, and then mixed. We adjusted the statement accordingly (line 120f)**

Line 125: .. between defecation and freezing, or? For stability it is less important when the sample was collected!

**Between defecation and freezing, we adjusted the text accordingly (line 127)**

Line 128-129: I can imagine what was performed, but wording is unclear.

**The sentence has been rephrased (line 130ff)**

Line 142: via a cortisol enzyme immunoassay…. against cortisol-3-CMO:BSA.

**The sentence has been corrected (line 144f)**

Line 149: “Suited” is better than “reliable”. How would you define the latter? Most important is an EIA, which can monitor expected increases/decreases in adrenocortical activity well.

**Corrected (line 151).**

Line 151 onwards: Four different EIAs were tested – please add EIA to each of the descriptions (e.g. 11-oxoaetiocholanolone I EIA) - I guess GB-spelling is chosen, so aetiocholanolone is correct.

**The sentence has been corrected (line 154ff)**

Lines 156-157: not possible to write by [number] – need to write the authors and then [].

**The sentence has been corrected (line 158ff)**

Line 159: delete “fGCM”

**The sentence has been corrected (line 161)**

Line 175-176: Peak fGCM concentrations following ACTH administration were expressed as percentage increase above baseline concentrations.

**The sentence has been corrected (line 177f)**

Line 183-186: How many samples per animal were present (I guess only baseline samples were used)?

**The missing information was added (line 186ff). Only baseline samples were used for this part of the study.**

Line 187: post defecation.

**The sentence has been corrected (line 190)**

Line 188: mean value … (no hormones in the faeces).

**The sentence has been corrected (line 191)**

Line 188/189: Unclear what the authors mean here? Why distribution?

**The sentence has been rephrased (line 191ff)**

Line 195: ..occuring

**The wording has been corrected (line 198)**

Line 209: enzyme immunoassays (EIAs)

**The sentence has been corrected (line 212)**

Table 1: The two 11-oxoaetiocholanolone have a higher increase – so why was the 5α-pregnane-3β,11β,21-triol-20-one EIA chosen? What means a “more stable” baseline? How is this made objective?

**As stated in the manuscript, all four assays tested revealed an overall increase in fGCM concentrations above 100% post ACTH injection and thus appear suited for monitoring alterations in fGCM concentrations in sows. Although the two 11-oxoaetiocholanolone EIAs tested show a higher overall increase after ACTH administration compared to the 5α-pregnane-3β,11β,21-triol-20-one EIA, the pre-injection baseline as well as the post-injection peak values for the three animals tested were most comparable when using the 5α-pregnane-3β,11β,21-triol-20-one EIA. We further knew that with at least the 11-oxoaetiocholaonolone I EIA, fGCM concentrations were not stable after defecation (a 175% increase after 4 h, 375% after 24 h) (Möstl et al. 1999). That’s why we finally decided to continue with the 5α-pregnane-3β,11β,21-triol-20-one EIA.**

**We adjusted the related statement which now reads ‘However, the 5α-pregnane-3β, 11β,21-triol-20-one EIA showed the most comparable baseline and peak values for all three animals and was chosen for the analysis of the remaining faecal samples.’ (line 207ff)**

Line 213: (range: 215.7…) – but the 205.7 given in Table 1 is lower, why that?

**Once the decision was made to continue with the 5α-pregnane-3β, 11β,21-triol-20-one EIA, the entire ACTH sample set (including the samples from the three animals used for EIA selection) were (re)analysed, which resulted in slight alterations in pre-injection baseline and post-injection peak values for the three animals already tested.**

Fig 2 (and the same for Fig. 1): How were those 4 chosen (of course this makes someone curious about the other 2 ones (especially as Möstl et al., 1999 found even higher increases in some pigs, but no expressed ones in others). Please show all 6 pigs.

**Only the individuals injected with Synacthen are shown in this figure, not the control individuals injected with saline. We added another figure depicting the two profiles of the control animals.**

Line 232 (235) – what does “V” mean?

**The V value is the sum of signed ranks (typically referred to as W). It has now been replaced by the corresponding Z value (lines 235, 238).**

Line 248: first time of “GIT” – spell it out!

**Thanks for pointing that mistake out, we now refer to ‘gastro intestine transit (GIT) systems’** **(line 251)**

Line 249: add [8] to [14]

**The reference has been added (line 252)**

Line 250: “suited” is better than “sufficient”

**The sentence has been corrected (line 253)**

Line 251: [20] they also used a dexamethasone suppression test, and the assay was found unsuited, based (at least partly) on the fact that the suppression was not well reflected in fGCM levels.

**The information has been added (line 254ff)**

Line 250/251: Those studies did not perform ACTH test or biological validations “to measure fGCM concentrations” – please reword.

**The sentence has been rephrased (line 254ff)**

Line 256: .. a cortisol assay (using an antibody against cortisol-3-CMO:BSA) …

**The sentences has been corrected (line 260f)**

Line 259: barrows? Did you mean “farrows”?

**Barrow can be used to describe castrated male pigs**

Line 263: no hyphen in case of enzyme immunoassay. “glucocorticoid metabolites” – and also reword the rest (see comments above).

**The sentence has been rephrased (line 269)**

Line 265-267: Please reword. I wonder, but cannot find a sustained (up to 48 h) peak in Fig 2?

**The sentence has been rephrased (line 270ff)**

Line 268: baseline levels between 221.5 and 246.3 ng/g: I’m confused, as this is the third interval given for the baseline levels (Tab 1; line 213), and all are different?

**We sincerely apologize for the confusion. As explained above, the different values stated in Tab1 compared to line 213 result from a re-measurement of the sub-sample set originally used for the assay screening. The values mentioned in line 268 are only referring to the animals treated with ACTH and not all six study animals (as referred to in line 213). To assist the reader we are now referring to all six animals here as well (line 272ff).**

Line 269 – repetition of line 266 (180% increase)?

**The sentence has been corrected (line 272ff).**

Line 270: delete one full stop.

**The mistake has been corrected (line 275).**

Line 272: .. and the levels did not reach baseline after 24 hours [32].

**The sentence has been corrected (line 277f).**

Line 275: “fGCM recovery time”?

**The sentence has been rephrased (line 279ff).**

Line 276: .. and the appearance of their metabolites in the faeces [33].

**The sentence has been corrected (line 281f).**

Lines 283-286: I suggest adding “other species” and “different measurement method” as the most likely reasons for the observed differences.

**The information has been added (line 289f).**

LINE 288: “were” instead of “was”

**The sentence has been corrected (line 294).**

Line 294: .. when evaluating stressors in pigs using saliva as sample matrix.

**The sentence has been corrected (line 299ff).**

Line 296: ..might be a more suited..

**The sentence has been corrected (line 302)**

Line 299 “steroid concentrations”

**The sentence has been corrected (line 305)**

Lines 298-315: this is a rather long passage dealing with the stability experiment. I suggest focusing on domestic livestock and shortening it (e.g. the sentences lines 307-310 can be easily deleted without losing much). As mentioned before, I doubt there is much application in pigs for collection in remote locations and long sample transport.

**The paragraph has been shortened (lines 304ff)**

Line 322 onwards: I would prefer more straight forward applications in immature pigs (too many potential things/possibilities) – but see also further comments below!

**In light of the aligned comments below we rephrased the conclusion section to address the various points raised**

Lines 326-327: Don’t think that this statement is true. Your aim was validating such a non-invasive tool, but the importance must be beyond the tool.

**The sentence has been removed**

A clear statement about the limitations/weaknesses of the study needs to be added here. Only female, immature pigs were used. Thus results don’t guarantee a broad application of the method in pigs, because reproductive status (age) and sex are serious confounders in fGCM analyses, and thus their influence needs to be elucidated. That clearly limits the application of the method!

**We agree with the reviewer and added a statement concerning the limitations (line 318ff).**

References: Not all words should be capitalized in the title [correct for 6, 9, 12,15, 17, 22, 28, 33, 36, 46].

**The references have been corrected**

Line 337: last accessed?

**The reference has been corrected (line 341).**

Line 346: delete the 1 after cattle.

**The reference has been corrected (line 351).**

Line 379: Domestic Animal Endocrinology

**The reference has been corrected (line 390).**

Line 388: the journal is missing.

**The reference has been corrected (line 399).**

Line 393: “Suppl 2” is missing.

**The reference has been corrected (line 405).**

Line 395: Veterinary Research Communications

**The reference has been corrected (line 408).**

Line 452: “ethological”

**The reference has been corrected (line 457).**

Line 453: better: “last accessed” than “updated”.

**The reference has been corrected (line 459)**

Fig 1 and 2: Smaller symbols for the 4 pigs would make the picture clearer, also allowing adding the missing two.

**We have changed both figures accordingly and added additional figures with the control animals.**

Fig. 2. What about the animal which showed a peak in fGCM concentrations at the time of ACTH injection? Was there a known stressor occurring the day before? There seemed to be a smaller peak (ACTH related) present on Day 1 (based on the broader shoulder).

**Unfortunately we don’t have any data on this point and can only speculate about an additional stressor perceived on that day.**

Fig. 3: The y-axis should be scaled from 0 to 100. This is more correct and also better representing the message of only insignificant changes in FGCM levels over time. Myjor x-axis ticks every 8 hours would fit the intervals better and also help the readers who usually think in days (i.e. 24 h not in 10/20/30 h etc.). Legend y-axis: % of initial fGCM concentration.

**We have changed figure 3 accordingly**

Reviewer #2: This is a very well written article. It is clearly explained and well executed. The methods for validation are sound and support the findings. The authors have effectively identified a new method that can used to noninvasively measure adrenal activity in pigs. I have no edits on the manuscript and would accept it in its current format. Great work.

**We thank the reviewer for the encouraging words.**
