# Supplementary material for: Non-invasive monitoring of adrenocortical function in female domestic pigs using saliva and faeces as sample matrices
Source: PLoS One. 2020 Jun 25;15(6):e0234971. doi: 10.1371/journal.pone.0234971 (PMC7316308; doi:10.1371/journal.pone.0234971)
Supplement: Attachment — Submitted filename: Response to reviewers.docx [file pone.0234971.s002.docx]

**Response to editor and reviewers**

PONE-D-19-24903R1
Non-invasive monitoring of adrenocortical function in female domestic pigs using saliva and faeces as sample matrices

Dear Dr Wolf,

Thank you for submitting your manuscript to PLOS ONE. After careful consideration, we feel that it has merit but does not fully meet PLOS ONE’s publication criteria as it currently stands. Therefore, we invite you to submit a revised version of the manuscript that addresses the points raised during the review process.

- fix the methods descriptions

- compare saliva and faeces data

- fix minor issues with legends etc.

We look forward to receiving your revised manuscript.

Kind regards,

Henrik Oster, Ph.D.
Academic Editor
PLOS ONE

**We thank the editor for the opportunity to resubmit our revised manuscript. As suggested, we have addressed the comments from the reviewers and edited the manuscript accordingly if required.**

Review Comments to the Author

Reviewer #1: Thank you – the revised paper is significantly improved now. The authors have sufficiently addressed my points; thus, I think the paper is suitable for publication now. Only a few minor errors are left for correction (typos; etc… - see details below), before it should go to the production department.

**We thank the reviewer and hope we have addressed the remaining points raised.**

The hyphen of “enzyme-immunoassay” was only partly deleted – so there is now an inconsistent situation. Please also delete it in the remaining cases (lines 144 and 153)

**We have now corrected the spelling (lines 153 & 162)**

The 11-oxoaetiocholanolone is sometimes still present in its US-spelling (please change “e” to “ae”; lines 154 and 155)

**We have now corrected the spelling (lines 163 & 164)**

Thanks for adding data of the control animals (Fig. 2b). I’ve only one question left now here: The mentioned increase after saline injection (60% above baseline; lines 219/220) is present on Day 0 – when were the samples collected in relation to the injection time – I wonder if that really reflects the saline injection? If too close, I suggest deleting that sentence.

**We agree with the reviewer and omitted the statement**

Line 260: please delete the first “”an assay using”

**We have corrected the sentence (line 277)**

The problem with the capitalized title words is not fully resolved – please change all occurrences in refs 6.; 8; 10; 18 and 35 (correct only with the first word after a “:”).
Line 401: A typing error was introduced: “acpacity”.
Ref 33: “American”
Ref 34: The end was lost during revision; please add: Wiener Tierärztliche Monatsschrift. 2010;97: 259–262.

**We have now corrected the references**

Reviewer #3: The revised paper is well-written and the goals of the study are clear and well-justified. The results of the studies described will be of use to others that would like to implement non-invasive fecal glucocorticoid metabolite measures with domestic pigs. The methods appear sound, although there are some details that are missing or presented in a confusing manner that makes it difficult to interpret the significance of the results. There are important changes to address to help readers interpret the study design and results, however these should be relatively easy to address.

**We thank the reviewer for the valuable input and hope that our revised version sufficiently addresses the respective points raised.**

Introduction:
The background information is clearly presented and the motivation for the current study is clear. The study objectives are sound.

Methods:
Overall, the methods are well-explained and easy to follow. The different sub-studies are well-designed, and the sampling regime was good.

There are three sub-studies in the current work: (1) document post-ACTH and post transport salivary/fecal corticoid peak concentrations and timing, (2) compare morning vs. evening salivary/fecal corticoid concentrations, and (3) determine relative degradation of glucocorticoid metabolites in feces kept in ambient conditions. These three goals/sub-studies are not presented as distinct, separate studies in the methods and results, and this makes it difficult at times to elucidate the sample size and sampling/analytical methods associated with each result. One way to clarify the methods would be to provide salivary and fecal sampling times relative to each manipulation (e.g. ACTH, transport, time of day). This is well done for the degradation study, but difficult to understand for the ACTH/transport studies. Please also see comments about Results below.

**To assist the reader, we have now added more information concerning the timeline of events and sampling regime, and subdivided the methods section further into the specific parts of the study, namely the monitoring of transport and the effect of sampling time.**

One example of this confusion: there is disagreement in the description of when fecal samples were collected in the methods vs. how data are presented in Figure 2. In the Methods, the authors indicated that fecal samples were collected during the same 7.5-hour continuous window on a daily basis (8:00-15:30 h). However, in Figure 2, there are data points that indicate that samples were collected every 12 hours rather than every 24 hours (i.e. the data points after ACTH injection). This should be clarified in both the Methods and the Results. For example, the authors should indicate when ACTH injections were administered relative to fecal sample collections.

**We apologise for the confusion and have now added a more detailed description of the sampling in the methods section. We now also indicate that ACTH administration took place in the early morning of day 0 (line 103). We also want to point out that Figure 2 shows the median fGCM levels per day for each individual, and only on the 2 days after injection we show fGCM values of all individual samples to better visualize the time when the peak concentrations were found.**

In the above example, there is another concern because a peak in fGCM is more readily documented if samples are collected more frequently (as they appear to have been done post- vs. pre-ACTH injection). Specifically, when samples are collected more frequently, there is more chance to catch a true peak without averaging a peak sample with other lower-concentration samples collected during a wider sampling window. Thus, if the sampling regime became more frequent after the ACTH injection compared to before the injection, then there is some concern about methodological bias leading to a greater chance of documenting peak fGCM after vs. before ACTH injection. The authors should clarify this.

**We apologise for the misunderstanding, the sampling regime over the course of the ACTH challenge did not change, as samples were taken from all faeces voided between 8:00 and 18:00h. We now indicate that in the revised version of the manuscript (line 123). This should indicate, that there is no bias between the samples collected before and after ACTH injection.**

It is good to see that the authors compared multiple EIAs.
To compare morning vs. afternoon salivary cortisol and fGCM it would be more appropriate to compare corticoid concentrations from samples that are collected at more distinct time points. In the present design, the authors compare ‘morning’ to ‘afternoon’ corticoid concentrations by comparing corticoid concentrations from samples collected between 8:00-12:00 h (morning) vs. 12:01-18:00 h (afternoon). These are not particularly distinct time frames – the morning samples may consist of a majority of samples collected near 11:00 h while afternoon samples could be primarily from 12:30 h. To truly determine if there is a circadian rhythm, it would be best to compare samples that are collected at the beginning vs. the end of the active period. To truly analyze if there is a circadian rhythm in fGCM, the authors should compare mean fGCM from 8:00-12:00 h vs. 14:00-18:00 h, or better yet, compare fGCM from samples collected 8:00-10:00 vs. 16:00-18:00 h. As currently conducted, this analysis favors the authors hypothesis that fGCM measures will fluctuate less across the day compared to salivary cortisol measures.

**We agree with the reviewer that this is a valuable point, originally, we used fGCM values from samples for the ‘afternoon’ sample set collected between 12:45 and 18:00 for this analysis. However, when excluding samples collected before 16:00, the mean of the afternoon samples appears comparable to the mean including the early afternoon samples (235.6 vs 240.8 ng/g DW; W=5134, p=0.738). All morning samples were collected between 8:00 and 10:00am, and fGCM concentrations did not differ significantly regardless of comparing steroid values from the morning samples with those from the more broader (12:45 – 18:00) or stricter (16:00 – 18:00) afternoon sample set. We therefore decided to keep the currently presented approach. However, we are willing to exchange it with the one representing the more rigorous time bouts if requested.**

Were all animals equally represented in the morning vs. afternoon samples? This should be clarified.
The authors should indicate the sample size involved in each statistical analysis. And to indicate how often each animal was represented in each data set (e.g. was animal ID controlled for in analyses that included multiple samples from the same individual?).

**All individuals are represented equally in morning and afternoon samples as indicated in the methods section (line 132ff & 194ff). 6 morning samples were compared to 6 afternoon samples from the respective days. It is therefore not necessary to control for individual bias within the analyses.**

In the current study, blood samples were not collected to allow for a comparison of fGCM concentrations to blood cortisol concentrations as a validation step. However, both saliva and feces were collected from the same six pigs. Can the authors include an analysis to compare individual salivary and fecal corticoid measures? In other words, were pigs that had high baseline or post-ACTH salivary cortisol levels the same pigs that had high fGCM concentrations at these times? This is not necessary, but it would provide additional validation of the fGCM method used in the current study.

**Salivary GC concentrations, similar to GC concentrations in blood, provide a valuable insight into GC fluctuations over a small timeframe by indicating what is going on in the organism seconds to minutes before the sample was collected (Anestis 2010). They are therefore also prone to additional factors like diurnal rhythm of GC production. Faecal samples on the other hand provide a more cumulative picture of GCM concentrations, providing an insight into a time depth of up to 40 hours, depending on the size of the organism (Touma et al. 2004; Anestis 2010; Behringer & Deschner 2017). As animals, even if kept under standardised conditions, might perceive various occurring stressors differently, the cumulative signal depicted in fGCM concentrations will most likely differ from that point-in-time GC value revealed in a saliva sample. We therefore don’t think that a comparison of salivary GC and fGCM concentrations would be reasonable and thus haven’t included any additional information in the revised version of the manuscript. However, we did a respective comparison of individual baseline sGC/fGCM levels, and as expected the animals with the highest or lowest sGC concentrations do not have the highest or lowest fGCM levels. Interestingly, and another possible reason for the mismatch might be that especially the fGCM baseline values only vary minimally between the individuals (216 – 246 ng/g DW) and could thus be considered equal.**

Results:
Please include sample size in all reported result that included an average or median value (in particular for ‘Effect of Collection Time’ and ‘Stability test for fGCM concentrations’ results).

**The sample size per individual for the analyses of the collection time is indicated in the methods, we now added the total numbers (line 198). The sample size for the stability test is 24 as already indicated in the methods (line 138).**

Line 216: Clarify that the results in this section are from the 5α-pregnane-3β,11β,21-triol-20-one EIA. In addition, explain how the average baseline fGCM level was calculated. How many samples were included from each female? Were individual female means calculated on each day or across multiple days, and then an average taken from all individual female averages? Please indicate how differential sampling from individual females was accounted for in the calculations, or at least provide information on how much each female contributed to the sample size.

**The EIA name has been added to the sentence. ‘To establish a baseline individual median fGCM concentrations were calculated using pre- ACTH administration samples’ (line 185). 9 samples per individual (collected 5 days prior to ACTH injection) were used to establish individual baseline levels. We have added more information (line 99ff).**

Figure 2 legend: Indicate that values in figure are from the 5α-pregnane-3β,11β,21-triol-20-one EIA.

**The information has been added to the figure legend (line 234)**

Line 241: “Within the first hour,” should read “Within the first hour at ambient temperature,”

**The sentence has been corrected (line 255)**

Discussion:
The authors reference two papers that previously used fecal corticoid measures in domestic pigs with ‘mixed results’. Given the relative importance of these prior papers relative to the current manuscript, the authors should tell us more about why the methods used in the current study led to clear results, while the prior work led to mixed results. What were the mixed results in prior studies? What methods did they use? And why were those prior studied unsuccessful while the current study was successful? These are important aspects that should be clarified.

**Additional information concerning the used methods and outcome in these studies has been added to the discussion (line 270ff)**

Otherwise, the Discussion appropriate compares results of the current study to results from other relevant studies.
